# The Control of Cultural Heritage Microbial Deterioration

**DOI:** 10.3390/microorganisms8101542

**Published:** 2020-10-07

**Authors:** Francesca Cappitelli, Cristina Cattò, Federica Villa

**Affiliations:** Department of Food, Environmental and Nutritional Sciences, Università degli Studi di Milano, Via Celoria 2, 20133 Milano, Italy; cristina.catto@unimi.it (C.C.); federica.villa@unimi.it (F.V.)

**Keywords:** biodeterioration, natural and synthetic biocides, nanoparticles, UV irradiation, gamma radiation, laser cleaning, heat shocking, microwaves, dry ice treatment, biological methods

## Abstract

The microbial deterioration of cultural heritage includes physical and chemical damage as well as aesthetic alteration. With the technological advancement, a plethora of techniques for removing unwanted microorganisms have opened up new opportunities for microbiologists and conservators. This article reviews the most applied, up-to-date, and sustainable techniques developed for the control of cultural heritage microbial deterioration presenting noteworthy case studies. These techniques include chemical methods, i.e., traditional biocides and nanoparticles; physical methods, such as mechanical removal, UV irradiation, gamma radiation, laser cleaning, heat shocking, microwaves, and dry ice treatment; and biological methods, such as natural molecules with biocidal activity, enzymes, and microorganisms. The application of control systems requires the comprehension of their behavior toward the unwanted microorganisms and possible interactions with the heritage materials. This overview shows also the control methods drawbacks for the purpose of creating awareness in selecting the most suitable technique or combination of techniques.

## 1. Introduction

Biodeterioration involves a combination of physical and chemical damages together with aesthetic alteration to cultural heritage. The term biodegradation is not used here, as according to some researchers, it involves degradation in a positive or useful way, i.e., rendering a waste material more useful or acceptable [1]. Due to technological innovations, a plethora of techniques and their combination to remove unwanted microorganisms have opened up new opportunities to both microbiologists and conservators [2,3]. Here, we present an overview of methods and their drawbacks to control the biodeterioration of heritage materials, with a special focus on outdoor stone heritage (Table 1). This review is important, as among a range of traditional and new methods, scientists and conservators do not always have clear pros and cons of using one technique rather than the other.

Before removing microorganisms, those causing biodeterioration should be clearly identified. Indeed, when the efficacy of treatment is uncertain, e.g., not targeting the actual biodeteriogens as these are not known, any treatment should be carefully considered or avoided [4]. Studying the microbial community of the Preah Vihear temple in Cambodia using DNA and RNA sequencing, Meng et al. [5] showed that the active community revealed by RNA sequencing was different from the whole community revealed by DNA sequencing. Thus, active members in the community need to be studied based on RNA rather than DNA to provide significant information for the monument’s conservation. In addition, as detected by metabolomics, microbes growing on different substrata produce different amounts of metabolites, e.g., organic acids, so that the mere presence of a biodeteriogen identified by DNA sequencing is not necessarily translated into a real biodeteriogen of a cultural heritage substrate [6]. For a recent overview of the use of -omics tools for assessing the biodeterioration of cultural heritage, the reader can see the review by Gutarowska [7]. DNA and RNA sequencing together to metabolomics will generate knowledge concerning the biodeterioration mechanisms caused by microbial communities and will help select appropriate prevention and control strategies.

**Figure 1:**
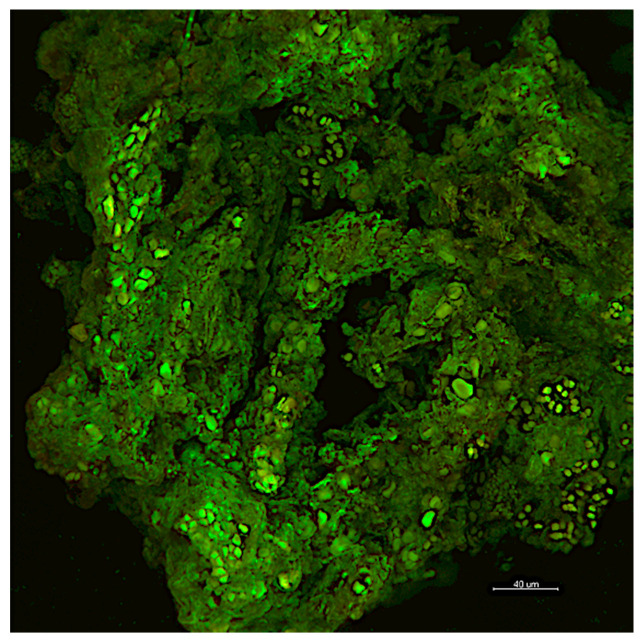
Confocal laser scanning imaging of biofilm growing on a Candoglia marble after a chemical cleaning (5% benzalkonium chloride followed by hydrogen peroxide) combined with a mechanical treatment (mechanical brushing and wet brushing) as previously reported by Villa et al. [12]. The green signal corresponding to Syto9 stain, which binds to DNA, appeared to be spread across the mineral surface. This result indicated the presence of intracellular material on the marble, which may suggest that dead cells were not completely removed after the cleaning treatment. The scale bar is 40 µm.

In the past, prior to applying control treatments, biodeterioration effects were not thoroughly analyzed [8]. Several authors have reported high numbers of microorganisms in decaying stones, and they concluded that microorganisms cause the deterioration [9]. However, an alternative and likely explanation could be that the decayed stones supplied a preferred habitat for microbiological growth [9]. Moreover, despite being extensively colonized by biofilm, the cathedral of Monza, Italy, was much more affected by chemical–physical deterioration rather than biodeterioration [10] (Figure 1). Indeed, not even endolithic growth can be definitely associated with biodeterioration. In a study on Wyoming canyon sandstone engraved with ancient petroglyphs, it was proven that under dry conditions, the gaps between grains seemed to be sufficiently large to accommodate lichen hyphae without exerting undue pressure on the rock structure [11].

As a consequence of the previous issues, control treatments should be adopted only after an in-depth scientific study proves it is really needed and is the best method available with respect to the target microorganisms.

## 2. Chemical Methods

### 2.1. Traditional Chemical Biocides

Biocides have been used for any kind of cultural heritage material both indoors and outdoors. The Biocides Directive 98/8/EC on placing biocidal products on the market gives the framework for biocide policy in the EU. In this directive, active substances are evaluated, and the decision on their inclusion into Annex I of the directive shall be taken at the EU level. Inclusion in Annex I may be denied if there are less harmful and, therefore, more suitable substitutes available for the same purpose. Nugari and Salvadori [4] stated that the only effective gas against insects and fungi for fumigation (the use of toxic gases in airtight boxes) was ethylene oxide, which has been banned in several countries due to carcinogenic and mutagenic features. However, more recent research [13] proved that vaporized hydrogen peroxide disinfection for new and historical cardboard at the Auschwitz–Birkenau State Museum in Oświęcim, Poland, has comparable effects to that of ethylene oxide.

If a biocide approach is planned to control microbial growth on a heritage surface, in situ pilot tests to calibrate biocidal treatments on the particular study case (species, site) often need to be run. Studying five biocides (namely, BiotinR, BiotinT, DesNovo, Lichenicida 464, and Preventol RI80) against lichens, Favero-Longo et al. [14] showed that different biocidal products and application methods have different efficacies against each species tested. In addition, the efficacy of a biocidal treatment against a lichen species is not consistent across different heritage sites.

Despite their toxicity, traditional biocides are still largely employed to contrast biodeterioration [15,16]. However, biocidal treatments have a brief duration and must often be repeated frequently, creating a repeated threat to the heritage material and the environment [17]. In addition, repeated biocidal treatments can cause resistance in target biological agents, and they can modify biofilm structures favoring the growth of more harmful biodeteriogens [12,18]. Biocide application has indeed caused damage to non-target organisms. According to Faimon et al. [19], in 1981, bats died in the Javořičko Caves (Czech Republic) because of hypochlorite cleaning.

As an alternative to traditional biocides, dimethyl sulfoxide (DMSO) in a solvent gel has been used as the active substance to remove biological colonization on marble and compared to biocides currently used in conservation [20]. DMSO solvent gel proved efficient at cleaning stone, and it was inexpensive, simple to employ, did not interact with pigments, and was therefore considered a practical treatment in comparison to commercial biocides. Importantly, although DMSO is generally considered to have low toxicity, Verheijen and colleagues [21] reported large-scale deregulations of cardiac microRNAs and smaller, but still extensive, effects on hepatic microtissues.

Recolonization on outdoor heritage is generally to be expected and depends on several factors, such as the antimicrobial agent employed and the way it was applied, the nature of the material and its state of conservation, the type and degree of colonization before the use of the biocide, and the micro- and macro-environment [18]. In 1963, regarding the ‘maladie verte de Lascaux’, the scientist Dobat believed that fighting algal invasion with chemicals was, in the long run, doomed to failure because it was impossible to kill all algae and spores that were present in the cave, new microorganisms would always be introduced, and chemical treatments would be frequently needed with high risk of harming the painting [22]. The problems of using chemicals and recolonization have not been solved yet. The site of Feilaifeng, which includes Buddhist statues, was inscribed in the UNESCO list in 2011. The statues affected by biodeterioration were successfully treated with the biocides AW-600 and octhilinone, but recolonization was observed shortly thereafter [23].

There are two possible outcomes after the application of biocides: (1) no residues of the antimicrobial agents are left, and this avoids any undesirable effects on the heritage object; (2) the biocide residues are not removed, so recolonization of the surface is delayed as much as possible. Generally, the first outcome is widely desired, considering the unpredictability of the recolonization rate and the usual insufficient information related to commercial products [18].

The use of traditional biocides in heritage repositories and environments is increasingly discouraged because of their toxicity. Combining biocides with substances that do not kill microorganisms but affect their biofilm development has been proposed to control microbiological growth on cultural heritage, which reduces the use of toxic substances. In this respect, biodeteriogens from the wooden sculpture So It’s Come To This (1986) by Bruce Armstrong, at the University of Melbourne headquarters, have been treated with a nitroxide, a compound with antibiofilm activity similar to nitric oxide (NO), which is a molecule involved in the modulation of cell-to-cell communication that is able to stimulate bacterial dispersal [24,25,26]. A 24 h treatment with 50 µM nitroxide followed by 2 h treatment with 0.001% *w*/*v* (a concentration much lower than that generally handled (2% *w*/*v*)) benzalkonium chloride successfully removed sessile growth. In the EU project BIODAM (Biofilm Inhibitors of Damage on Materials), biocides have been paired with permeabilizing agents, pigment and exopolymer inhibitors, and photodynamic agents in order to increase microbial susceptibility, thus reducing the amount of biocide employed [27]. The photodynamic agents nuclear fast red and methylene blue, tested under laboratory conditions in combination with hydrogen peroxide in BIODAM [28], showed the potential to kill the cyanobacteria *Synechoccus leopoliensis* on stone specimens and degrade under visible light, therefore not causing discolouration of the substrate. In particular, combining methylene blue with hydrogen peroxide resulted in a 40% reduction in the fluorescence.

### 2.2. Nanoparticles

Nanoparticles (NPs) display unique physicochemical properties including an ultra-small size, large surface-to-mass ratio, and a peculiar reactivity with organisms, and they are appealing to use both for organic and inorganic materials. Some NPs composed of silver (Ag), copper (Cu), titanium dioxide (TiO_2_), or zinc oxide (ZnO) display interesting biocidal features [17]. Nanocomposites are materials made by different NPs. Multiple mechanisms of action are associated with NPs and nanocomposites, including disruption of the cell wall and the plasmatic membrane, the inhibition of protein synthesis and DNA replication, and the enhanced oxidation of cell components and compounds. Interestingly, several bacteria and fungi are able to produce metabolites involved in the synthesis of various NPs. The advantages and drawbacks of using nanocomposites are reported in a recent review by Omanović-Mikličanin [29].

Gutarowska and colleagues [30] found that the most common fungi found in six museums and archives in Poland were *Aspergillus* (potentially allergenic and toxic species), *Penicillium*, *Cladosporium*, *Alternaria*, *Mucor*, *Rhizopus*, *Trichoderma, Paeciliomyces, Aureobasidium, Botrytis*, and *Chrysonila*. They demonstrated that a concentration of 10–100 nm nanosilver particles at 90 ppm was effective in removing the microorganisms present on the surface of the documentary heritage works. Importantly, the silver NPs antimicrobial mechanism of action is not fully elucidated yet. Two nanocomposites based on silver and titanium dioxide NPs had been successfully used as biocides for masonry materials [31]. However, their durability was investigated by the same team later studying the in-depth penetration of the nanocomposites by Laser-Induced Breakdown Spectroscopy (LIBS) [31]. Notably, LIBS detected nanoparticles under the surface despite the limited application of nanoparticles. Silicon and acrylic stone consolidants were functionalized with AgNPs, which were synthesized via volatile metabolites produced during the aerobic growth of *Nesterenkonia halobius*, and they suppressed or prevented the development of *Streptomyces parvullus* and *Aspergillus niger* [32]. van der Werf and colleagues [33] reported the bioactive properties of Estel1100/ZnO nanocomposite material against the fungus *Aspergillus niger* in in vitro experiments. The results indicated that a tenfold higher concentration of zinc oxide nanoparticles, compared to silver nanoparticles, can be utilized in the matrices without affecting the color of the stone substrate, exerting a long-lasting biocide activity on the substrates.

Many papers have proposed using NPs to prevent rather than control biodeterioration. Franco Castillo and colleagues [34] investigated magnesium oxide nanoparticles to protect 18th century papers from fungi without altering the appearance of the samples. Zarzuela et al. [35] developed, via a sol–gel route, CuO/SiO_2_ nanocomposites as protective coatings for built heritage. They studied a reference laboratory bacterium and yeast on limestone slabs under laboratory conditions and proved that the release of Cu^2+^ ions is the most likely mechanism for the biocidal effect. Soria-Castro et al. [36] evaluated two types of NPs based on calcium zinc hydroxide dehydrate and zinc oxide, and they were used as antimicrobial stone protective coatings to prevent the growth of bacteria and fungi in vitro. The authors stated that the results were difficult to compare with similar literature, as the method of synthesis, size, shape, and surface change of the evaluated NPs as well as the assays used to detect antimicrobial activity were different.

## 3. Physical Methods

### 3.1. Mechanical Removal

Traditional mechanical methods remove biodeteriogens on organic and inorganic objects with tools such as brushes, scalpels, spatulas, scrapers, air abrasives, high-pressure blasting, low-pressure washing, and vacuum cleaners. Phototrophic biofilms should be completely dry before any mechanical cleaning, because dry crusts often readily detach from the materials and can be easily removed by using brushes, sand blasting, or low-pressure washing [37].

Generally, mechanical methods have the benefit of not using additional compounds in the heritage substratum. For instance, Lee et al. [38] treated a standing Buddha engraved on a shale wall (South Korea) by dry cleaning followed by wet cleaning using distilled water. The researchers observed the removal of persistent lichens by soaking them in distilled water and using soft brushes and wooden knives to completely remove them. Sanmartín et al. [39] tested the efficacy of the dry brushing method, among other chemical methods, in removing an algal biofilm formed on a granite-built historical monument in Galicia (Spain). Color data showed no evidence of recolonization by phototrophic organisms after one year on all the trial cleaning areas. On the basis of these results and concerns about the potential toxicity of the chemical products tested, the researchers recommend removing biofilm from the cloister wall by using mechanical treatments such as brushing.

In indoor environments, airborne microorganisms can deposit on and colonize artworks. Powdery fungal colonies contain high amounts of spores and, therefore, are sources of contamination for other objects because air easily transfers the spores. In such cases, mechanical cleaning using a vacuum cleaner equipped with high-efficiency particulate arrestants (HEPA filters) is a suitable procedure to remove most hyphae and spores in both the air and on surfaces [40]. Settled airborne dust has been used as a surrogate for airborne exposure in laboratory-scale studies that explore indoor microbes [41]. Recently, the efficacy of mechanical methods, including dusters and vacuum cleaners, in removing dust deposits at the National Archives in the UK has been evaluated [42]. To this end, three document boxes each containing replicated files were positioned side-by-side on a trolley to simulate the storage of boxes on shelving in the laboratory. The National Archives’ Collection Care Department developed a method that used UV-fluorescing powder to mimic the movement and dispersal of dust during experimental cleaning and handling scenarios. Among all the methods tested, vacuuming was the most efficient, because it permanently removed UV-fluorescent powder from the card surface, and it minimized powder redistribution.

Despite some advantages, mechanical removal techniques may damage the substrate, e.g., the mechanical removal of cave microorganisms with water and brushes damaged the fragile crystal structures of speleothems, high-pressure vapor ruined tiny flowstone forms [43], and the use of high-pressure water blasting can also push microorganisms deep into the heritage material. Furthermore, when microorganisms grow endolithically penetrating pores, fissures, and cracks, it is hard to reach them mechanically, and residues in the form of single viable cells or whole colonies are a source for a rapid substrate recolonization [37].

A commercial product called Hydrogommage—a sand blasting, low-pressure (0.5–1.5 bar) air/water mixture with SiO_2_ particles (0.5–0.1 mm)—was applied on granite specimens to remove thick phototrophic biofilms composed of the green algae *Trebouxia* sp. and the cyanobacteria *Gloeocapsa* sp. and *Choococcus* sp. under laboratory conditions [44]. Although the treatment efficiently removed the biofilms, it caused textural changes to granite including an increase in roughness and microfissures [44]. The marble statues in the gardens of the National Palace of Queluz, Portugal were subjected to a grit-blasting treatment to remove biological colonization [45]. Mechanical treatment increased the surface roughness as compared to the uneven and pitted surface left behind by the chemical cleaning methods. The surface roughness resulting from the cleaning intervention affected the subsequent microbial recolonization of the marble [45].

The mechanical removal of biofilms by scrubbing and washing the dead biomass after a biocide-based treatment is a common practice adopted by conservators (*inter alia* [10,46,47]). In some cases, conservators remove the dead biomass immediately after treatment, while in other cases, they leave the objects untouched for several months and then lightly brush to remove any detaching remnants, reducing the intervention to a minimum [45]. However, as already said, leaving the dead biomass in place might provide nutrients for spores and microorganisms to develop (Figure 1).

### 3.2. UV-C Irradiation

UV-C irradiation has been applied to stone surfaces.

Regarding the ‘maladie verte de Lascaux’, in the 1960s, in addition to the modification of the physicochemical parameters of the Cave (CO_2_, temperature, humidity), the scientist Dobat envisaged the use of appropriate filters to monitor the quality of light as well as ultraviolet rays to kill the phototrophic microorganisms [22].

Species able to live in aquatic and terrestrial habitats, e.g., cyanobacteria, have adopted survival strategies when exposed to UV light. The terrestrial species of *Tolypothrix*, *T. byssoitka*, isolated from the stone surface of the Sun Temple, Konark, India, could survive UV-C irradiation up to 1 day, whereas the aquatic *Tolypothrix* UU 2434 was killed after exposure to UV-C for half an hour [48]. Prolonged exposure to UV-C radiation causes damage to photosynthesis-killing cyanobacteria (PS II is highly sensitive due to the absorbance of their proteins) and eukaryotic algae [49].

Borderie et al. [50] proved that UV-C treatment was effective in contrasting algal biofilm in a cave located in northeastern France (Moidons Cave) for a period of more than one year. In contrast to the use of chemicals that are dispersed by aerosol, the authors claimed that the method was able to work only on the target area [51]. In addition, Pfendler et al. [52] claimed that in situ UV-C treatment in comparison to chemicals is efficient, faster, cheaper, and environmentally friendly. However, due to layers of cells arranged upon each other and to the poor penetration of UV radiation, multiple radiation treatments might need to be employed to treat biofilms [52]. As some fungi are resistant to UV-C due to pigments such as melanin, they can proliferate after the death of phototrophic cells upon the realized organic matter. This is a drawback that should be taken into account when this methodology is employed [52].

Repeated short-term exposure of the planktonic *Nostoc* sp. PCC 9104 to Biotin T^®^ and UV-C irradiation at 254 nm under laboratory conditions showed that the microbial growth was more affected by the biocide [53].

Finally, some precautions are needed before the application of UV-C treatment, including closing the site to avoid exposing visitors or heritage personnel, using a timer to limit exposure when handling, ensuring that no animals are present in the area to be treated, and that treatment does not affect inorganic compounds, but it can be deleterious for organic compounds [49,54].

### 3.3. Gamma Radiation

Gamma irradiation has been used for its consolidation and biocidal effects mainly on organic materials [55]. Both insects and microorganisms can be killed by gamma radiation, but the dose must be adapted to the targeted organism and must be below the threshold of tolerable depolymerization [56]. Kantoğlu et al. [57] demonstrated the efficiency of radiation treatment at 6 kGy to control insects and microorganisms present in the documents stored in the Ottoman Archives In Istanbul.

In contrast to UV radiation, gamma radiation can penetrate deeply into objects; therefore, the whole object will experience the applied inhibition dose [58]. The counterpart to this feature is that the object is not protected against a future attack. In addition, Cortella et al. [59] claimed that transparent objects, such as glass and gemstones, may be adversely affected by gamma irradiation.

An outstanding early treatment by gamma irradiation was the mummy of Ramses II in 1977 [59]. The mummy was under biological attack, in particular by fungi. After irradiation, the mummy has been displayed in the Cairo Museum in a sterile showcase to avoid contamination.

An oil painting by a contemporary artist was used as an experimental model to evaluate the effects of gamma treatment on inhibiting biological attack to paintings [60]. Negligible spectral and chromatic alterations were observed after treatment by Fourier transform infrared (FTIR) and FT-Raman spectroscopy and CIELAB color data, respectively. Strains of *Streptomyces* isolated from ancient Egyptian paintings were killed using gamma irradiation without causing discoloration to the paintings or affecting the binding media mechanical properties [61]. Similarly, no negative effects of gamma radiation were observed on the texture and color of parchment for the studied doses able to combat biodeterioration phenomena [62]. Samples of silk and wool fabrics were artificially aged and then irradiated with 10 and 25 kGy gamma-ray doses [63]. Geba et al. [63] claimed that increasing the irradiation dose above 10 kGy had drastic effects on the loss of elasticity and the mechanical resistance of the tested specimens. In another study on fabrics (wool, linen, silk, and cotton), it was proven that all specimens became discolored by exposure to gamma irradiation from 0.5 to 25 kGy, and this change in darkness was more evident in samples dyed with natural pigments [64].

Choi et al. [65] investigated gamma irradiation (dose 5 kGy) to control fungal contamination by *Aspergillus niger, Penicillium verruculosum*, and *Trichoderma viride* on a Korean wooden cashbox stored in a museum. As after two months no fungi were detected, it was claimed that gamma irradiation can be applied for the successful inhibition of fungal growth on wooden objects. Gamma irradiation is also a well-established, low-cost treatment used for the decontamination of paper-made objects of cultural heritage. The irradiation conditions for treating the highly resistant secondary colonizer *Cladosporium sphaerospermum* and the naturally occurring mycobiota retrieved on paper samples were evaluated in in vitro experiments [66]. Untreated and inoculated samples of paper were irradiated with doses commonly applied to cultural heritage objects, as well as significantly higher doses, at two dose rates that differed by two orders of magnitude. The results indicated that at the irradiation dose of 7 kGy, a reduction in the mycobiota was obtained. Thus, re-evaluating the recommended dose of 8 ± 2 kGy was suggested.

### 3.4. Laser Cleaning

In the last decade, this technique has been amply studied on outdoor stone surfaces due to its advantages over traditional cleaning methods: it is controllable, selective, contactless, and environmentally friendly [67,68]. The main drawback is that every study case needs to be optimized.

The Nd:YAG laser (0.5 ms pulse, 5 J:pulse, 1 Hz) yielded a satisfactory surface lichen cleaning outcome on outdoor sculptures at the Seattle Art Museum but at a comparably slow rate [69]. In the research by DeCruz and colleagues [70], it was noted that the Er:YAG ablation of lichens on stone produced a continuous white flash peculiar for the biological target. The flash steadily decreased as the lichen material was discarded. Pyrolysis gas chromatography–mass spectrometry, infrared spectroscopy, and high-performance liquid chromatography demonstrated that destruction of the polysaccharide in the cell wall of the fungus was the source of the white flash. Additionally, after ablation, the absence of red fluorescence using fluorescence microscopy showed the destruction of the photobiont. In vitro experiments carried out by Speranza et al. [71] showed severe cellular damage both for the lichen *Verrucaria nigrescens* and fungi and algae grown endolitically after Nd:YAG laser treatment with an infrared wavelength (1064 nm, 5 ns). As the optical absorption at 1064 nm is generally low for the main constituent substances of biological growth, such as water, pigments, lipids, and other compounds, when using the Nd:YAG laser, Osticioli and colleagues [72] proposed using the second harmonic at 532 nm. The authors reported that 532 nm laser irradiation was significantly more effective than 1064 nm, and it can solve a larger set of problems related to biodeterioration under laboratory conditions. A black biofilm of variable thickness composed of algae and fungi, which penetrated into the pores of the granite material, was effectively eradicated with a Nd:YVO4 laser working at an ultraviolet wavelength of 355 nm and fluence ≥0.5 J/cm^2^ [73]. However, the authors noted that biotite was damaged by melting, even at fluences ≤1.5 J/cm^2^. The UNESCO Ethiopian Lalibela church façades attacked by a microbial community constituted by bacteria, fungi, and lichens have been treated with laser cleaning to avoid abrasive particles or chemicals, minimizing the risk of damaging the fragile stone [74]. Lichen encrustations on basaltic stone were removed with UV laser pulses (355 nm) at a fluence of 0.35 J/cm^2^. In this study, laser treatment at 1064 nm was not successful, because the high thermal energy caused the stone minerals to melt.

The lichen *Circinaria hoffmanniana* was removed from schist collected from the Côa Valley Archaeological Park (Portugal) using two different laser systems working in the IR regime: an Nd:YAG laser at 1064 nm and an Er:YAG laser at 2940 nm, with different pulse durations [67]. The results indicated that both lasers, Nd:YAG and an Er:YAG, damaged the lichen structure without completely extracting the microorganisms. Thus, the authors recommended the use of a Nd:YAG laser because it induced less intense physical changes than the Er:YAG laser.

Barreiro et al. [68] investigated in vitro the effects of three different wavelengths (355, 532, and 1064 nm) of an Nd:YAG laser applied in removing a naturally developed sub-aerial biofilm from Vilachán granite, which is commonly used in monuments in the Northwest (NW) Iberian Peninsula. The results showed that a wavelength of 532 nm successfully removed the biofilm, but it induced the highest color modifications in the granite due to extraction of the kaolinite crackled layer and the Fe-rich segregations. The treatments at 355 and 1064 nm showed lower surface changes and residues of burnt organic matter that could induce recolonization. Overall, these findings suggest that the laser parameters should be optimized in order to avoid any side effects on the surface as a result of overcleaning.

### 3.5. Heat Shocking, Microwaves, and Dry Ice Treatment

Many microorganisms living on outdoor stone are thermotolerant (up to 65–70 °C) when dry but are thermosensitive when wet, and heat shock treatments on wet, metabolically active lithobionts cause a loss of membrane permeability and denaturation of proteins [75]. The method is simple, easy, and fully substratum-, operator-, and environment-friendly, and it employs plastic foils, thermal blankets, and infrared lamps [75].

The feasibility of the in situ thermal treatment was verified with several lichen species under laboratory conditions [75]. Six-hour treatment at 55 °C was able to kill the fully hydrated lichens. At 40 °C, thermal treatment damaged lichens and was combined with a concentration 10-fold lower than that normally used. In contrast to multicellular organisms such as lichens and mosses, Bertuzzi et al. [76] showed that in vitro heat shock treatment was not entirely effective against eukaryotic algae, likely favoring the growth of some resistant surviving cells.

Microwaves were applied to control biodeteriogens on both organic and inorganic materials. Cuzman et al. [77] proposed a fully portable microwave heating system to uniformly heat a 30 cm^2^ area with the black fungi *Sarcinomyces* sp., *Pithomyces* sp., and *Scolecobasidium* sp. isolated from cultural heritage. Three minutes at 65 °C was the assessed lethal dose, affecting both the mycelium and the fruiting bodies. Cerchiara et al. [78] successfully tested microwave heating at 58 and 63 °C on paper samples attacked by *Aspergillus versicolor* in order to remove the fungus. Further improvement included the combination of laser (532 nm) and microwave (2.45 GHz) heating treatment of biofilm from Carrara marble and was investigated to target both epilithic and endolithic growth [78]. Drawbacks of microwave heating are the presence of highly heated areas (hot spots) or areas with poor radiation caused by specific shapes. To overcome these problems, Pierdicca and colleagues [79] proposed a mathematical model predicting the distribution of heating power in heritage material to determine the optimal exposure conditions, exposure time, and power to be employed.

Giovagnoli et al. [80] investigated a dry ice blasting system for removing a biological black patina on stone surfaces of the Pyramid of Caio Cestio in Rome. The researchers concluded that the method could be used efficiently for cleaning biological black patina only if followed by a biocidal treatment and used on stone substrata in a good conservation condition.

## 4. Biological Methods

### 4.1. Biocidal Treatments with Compounds of Natural Origin

The search for alternatives to commercial biocides has led to several papers reporting on the application of natural products in the conservation. Natural biocides are considered safer for human beings and greener for the environment and have been used both for organic and inorganic materials [81]. Many of these products are derived from plants and could be employed in their pure form as well as crude extracts or as essential oils.

Tayel et al. [81] claimed that plant smoldering fumes could be used as an effective alternative to chemical fungicides, completely sterilizing the fumigated archival repository and leaving no modifications to color or surface structure of paper specimens. In addition, fumes did not stimulate the growth of other microorganisms, and residues did not remain in the treated materials, as fumes were extracted after treatment.

To date, the use of natural substances has largely focused on organic materials, such as wood and paper, in indoor environments [82]. However, inorganic materials have also been treated with these natural compounds [83]. Essential oils from *Thymus vulgaris*, *Origanum vulgare*, and *Calamintha nepeta* and their major components (thymol, carvacrol, pulegone) have been prepared within a hydrogel matrix (made of Gelrite, poly-vinylacetate, CaCl_2_, and Acemoll CC) to remove biofilm from a travertine wall of the Sapienza University in Rome [84]. Volatile extracts from *Illicium verum*, the flower buds of *Syzygium armoaticum*, *Quercus infectoria*, *Coptis chinensis*, and *Phellodendron amurense* were profitably tested with fungi isolated from biofilm on the World Heritage Yeongneung stone monument [82]. The best antifungal effects were shown for Eugenol isolated from clove extract. The volatile organic compound was used with the stable emulsifiers Tween and Span. The antibacterial eugenol was proven to downregulate YidC, which is a highly conserved bacterial protein that plays a vital role in membrane protein insertion. Furthermore, eugenol inhibits bacterial ATPases and the FtsZ assembly, compromising the bacterial cell division process [85].

*Thymbra capitata* essential oil was proposed for use against cyanobacteria and green algae on historical monuments [86]. The major components of the essential oil were the phenolic monoterpene carvacrol (73.2%) and its biogenetic precursors γ-terpinene (6.9%) and *p*-cymene (4.3%). The essential oil/water emulsion was stabilized with kaolinite, laponite caused the elimination of biodeteriogens from treated surfaces, and these good results were still maintained after four months.

*Origanum vulgare* and *Thymus vulgaris* essential oils showed strong antimicrobial activity in in vitro assays, which were subsequently confirmed in in situ applications on biofilm retrieved under the floor mosaic tesserae in the Greco-Roman archaeological site of Solunto, Sicily (Italy) [87]. According to the work, the antimicrobial activity of 15% *T. vulgaris* essential oil solution was enough to deeply impact biofilm development. The principal chemotypes identified were thymol and carvacrol. Recently, volatile compounds of the same essential oils, *O. vulgare* and *T. vulgari*, were used to combat the biodeterioration processes induced by the fungus *Aspergillus flavus* on wooden artworks [88]. To this end, the researchers developed ad hoc structures to expose wooden artifacts to the volatile compounds, avoiding any negative impact on the environment and on the operator’s health. The antimicrobial effect of thymol and carvacrol may result from a perturbation of the lipid fraction of the microbial plasma membrane, resulting in modifications of membrane permeability and in the leakage of intracellular materials [89,90].

Recently, Bogdan and colleagues [91] tested in vitro the antimicrobial activity of weeds extracts and their incorporation into waterborne paints to counteract bacterial biofilm formation. The extracts were obtained from *Raphanus sativus*, *Rapistrum rugosum*, *Sinapis arvensis*, *Nicotiana longiflora,* and *Dipsacus fullonum* weeds, being vegetables commonly used in traditional medicine as antimicrobial compounds. Findings reported the efficiency of the *Nicotiana longiflora*-based paint in inhibiting both *Escherichia coli* and *Staphylococcus aureus* biofilm formation.

Having a high relevance in stone conservation, the applicability of the lichen secondary metabolites usnic acid, norstictic acid, and parietin to control fungal, cyanobacterial, and algal growth on stone cultural heritage as well as their interaction with the white Carrara marble were evaluated in vitro with regard to putative chromatic alteration [92]. The natural compounds did not stain the stone specimens in a perceivable way.

In their review, Fidanza and Caneva [83] claimed that the efficacies of natural substances were extremely different across research experiments, as were protocols and doses, so best practices are still needed. In addition, the overview highlighted the lack of experiments discussing the interference of natural compounds with heritage materials. A further drawback of using chemicals of natural origin, such as oils and plant extracts, is that their composition can be substantially dependent on the harvesting season, geographical location, and other agronomic factors [93]. Additionally, the extraction methods influence the chemical composition of the extracts [94]. As a consequence, all natural mixtures must be thoroughly characterized in order to make the experiments repeatable. Alternatively, chemically synthetized products can be acquired from the market. Notably, some phytochemicals, such as essential oils, are costly. Therefore, a more affordable alternative to essential oils is to purchase the main pure synthetic counterpart that is also generally more stable [95].

### 4.2. Other Biological Methods

The following biological methods are environmentally friendly, low cost, and present low to no risk to human beings. These methods have been applied to different materials.

Valentini et al. [96] applied a new method based on the glucose oxidase enzyme to remove biofilms from travertine and peperino substrata of the Villa Torlonia in Rome (Italy). A comparative study was also performed to validate the enzyme-based cleaning procedure by using a saturated solution of (NH_4_)_2_CO_3_ and ethylenediaminetetraacetic acid (EDTA) and the enzyme lipase. Among all, the cleaning procedure using glucose oxidase showed the best results. Glucose oxidase produces H_2_O_2_ when glucose is present. Since the release is highly controllable, this method is rather safe for the substratum. Chitinases were also used to reduce the growth of filamentous fungi developed on word walls and canoes at the Cross Lake Bridge ruins in Xiaoshan [97]. Metal cations, such as calcium, magnesium, and iron, are involved in preserving matrix integrity. Antibiofilm formulations incorporating EDTA and other permeabilizers were efficient on in vitro biofilms [27].

Marin et al. [98] proposed a biological biocide named New FloorCleaner (containing spores of *Bacillus subtilis, B. megaterium*, and *B. pumilus*, claimed by the supplier to control the proliferation of other bacterial species) to remove biofilm on 20th century bricks. New FloorCleaner neither modified the surface according to the stereomicroscope observations and the material in depth according to the scanning electron microscope observations of cross-sections nor changed significantly the brick water absorption capacity and color. However, there was an increase in the conductivity of the aqueous extract in comparison to the control and, according to the researchers, this aspect should be investigated further in the near future. Interestingly, metabolites (not identified in the manuscript) produced by *Bacillus* spp. displayed around 100% antagonistic activity against *Fusarium oxysporium*, *Penicillium* sp. and *Alternaria* sp. and no lethality against brine shrimp and Swiss mice through the administration of a 5000 mg/kg acute dose [99]. Contrarily, Preventol^®^ caused acute toxicity with a 10-fold minor concentration dose administrated under the same conditions. The same *Bacillus*-based biocide was tested in vitro against the bacteria and fungi isolated from a 17th century easel painting attributed to Carlo Bononi, an Italian artist of the first Baroque period [100]. The results indicated that biological biocide inhibited the growth in vitro of all the isolated microorganisms (*Aspergillus* spp., *Penicillium* spp., *Cladosporium* spp., *Alternaria* spp., *Staphylococcus* spp., and *Bacillus* spp.), suggesting a broad-spectrum activity of the product.

Recently, a study carried out by Jurado et al. [101] on the speleothems of Nerja Cave (Spain) showed evidence that a form of natural biological control exists in caves, providing a new method to control phototrophic biofilms typical of lithic substrates such as monuments and walls of show caves. By studying the in vivo formation of plaques or spots in the biofilms where the phototrophic microorganisms disappeared, the researchers assumed the presence of predation processes operated by bacteria belonging to the genera *Bacillus* and *Lysobacter*, amoebas such as *Dactylopodida* and *Echinamoebida*, and insects. Hu et al. [102] demonstrated that fungi such as *Aspergillus allahabadii* may play a crucial role in the removal of preformed biofilms on sandstone at Bayon temple, Angkor Thom, Cambodia, offering a new way of removing biofilms on outdoor stone surfaces.

The potential exploitation of phages as biological control agents against wood-degrading microorganisms was investigated in vitro in the EU project BACPOLES [103]. However, while several bacteriophages were successfully isolated from microcosms and wood samples, the development of phage-based preservatives was not achieved. The main drawback in the development of phage-based wood preservatives relied on the isolation and cultivation of key bacterial players in the bioderioration of wood at laboratory scale, which is an extremely difficult and slow process. Finally, May et al. [104] presented proof of principle that algal types commonly found on the stones can be inhibited by viruses. To this end, the researchers isolated viruses with antialgal activity from mature biofilms, and then they tested the effectiveness of different viral treatments in laboratory pilot studies. Experiments were performed by using large-diameter limestone discs simultaneously inoculated with a growing culture of *Chlorella*, the predominant colonists on the investigated headstones, and the virus. It was successfully proven that on those limestone discs treated with the highest concentration of viruses, algal growth was not established. The authors concluded that a much wider range of viruses needs to be retrieved from the cultural heritage environment so that field trials against the natural algal populations identified can be performed.

In conclusion, biological technologies represent a promising approach to control cultural heritage biodeterioration and provide eco-sustainable alternatives, they are relatively easy to set up and improve, and they are harmless to humans and the environment [105]. However, further investigations are needed to monitor the safety and effectiveness of these approaches. In particular, it is important to (i) ascertain the absence of any possible interaction between the biological agent and the substrate, (ii) assess the persistence of treatment on surfaces over time with short-term and long-term surveillance, (iii) optimize the application methods and the number of applications, and (iv) evaluate the costs in comparison with chemical compounds or other common treatments.

## 5. Combination of Control Methods

There are many papers that compare the efficacy of different control methods on the same object or site. The comparison is extremely useful if for example considerations including environmental safety are taken into account [106].

The following literature proves that the combination of different control methods is often a successful strategy. Prior irradiation of the granite specimens with UV-B and UV-C light enhanced the cleaning efficacy of benzalkonium chloride [54].

Gamma irradiation has been used at sub-lethal doses followed by treatment with a biocide. This was the case of mural paintings in the Tell Basta and Tanis tombs exposed to various doses of gamma radiation (5, 10, 15, 20, and 25 kGy) and then treated with tricyclazole [107]. This combined treatment completely inhibited melanin production in the investigated biodeteriogens *Streptomyces* spp. Binding media (Arabic gum and animal glue) and pigments withstood every dose, with the exception of vermillion.

Laser cleaning can also be combined with other methods of control. According to Rivas et al. [108], mechanical weakening of the lichen by scalpel, followed by brushing, made cleaning easier with the Nd:YVO4 laser working at 355 nm. Additionally, the combination of laser and mechanical cleaning satisfactorily removed the darker lichen *Pertusaria amara* compared to the lighter *Pertusaria pseudocorallina*. The sequential use of laser irradiation and chemical treatment led to a complete damage of both photobiont and mycobiont cells, with no viability of the remaining lichen structures due to the chemical treatment [109].

## 6. Implications and Future Directions

Unfortunately, it is not possible to write a flow chart showing the key decisions to be made to select a control strategy, as many factors are to be considered for each case study that are known only to the researchers working at those projects. However, when specific aims have to be achieved beside the simple control, it is possible to suggest not choosing some methods, as these are unappropriated. Here, we present some of them. In case lithoid materials are not to be cleaned, laser cleaning and dry ice blasting are not an option at present. If environmental sustainability is an important issue, traditional biocides should not be used. When a low budget can be allocated, removal with specialized equipment is not recommended.

In order to prevent biodeterioration, the first approach includes the inspection and maintenance of heritage surfaces, reduction of biological particles in their environments, and active modification of the bioreceptivity of the heritage surfaces. To control biodeterioration, one must eliminate or destroy the already formed biofilm. In this overview, we have focused exclusively on control.

In this context, it is generally accepted that sessile microorganisms are more resistant to antimicrobial agents than their planktonic counterparts. This also is due to the development of resistant phenotypes upon adhesion to substrata and within the biofilm [110]. In investigating the effects of TiO_2_ nanopowder and TiO_2_ thin film, a decrease of one order of magnitude of *Pseudomonas aeruginosa* planktonic cells after 2 h and an almost complete removal of *P. aeruginosa* planktonic cells was observed. Contrarily, photocatalytic treatment with TiO_2_ film or TiO_2_ nanopowder did not affect *P. aeruginosa* sessile growth [111]. Green algae isolated in three caves grown in a mineral medium and inoculated on limestone specimens were subjected to UV-C irradiation [50]. This research reported that UV-C exposure was more deleterious when algae grew in liquid medium (planktonic growth) than on solid medium, simulating an in situ situation. Consequently, lab experiments are better performed on biofilms rather than planktonic cells, and therefore, the development of further biofilm lab models is needed [112].

Finally, rapid and routine analyses are essential to monitor the performance of the selected methods and to avoid undesirable effects on the substrate. Up to now, different studies use disparate methods to evaluate the success of a treatment even on objects made of the same material. The situation is further complicated by the fact that chemical, physical, and biological control methods could be used in combination. Several methods for assessing the effectiveness of a control strategy on stone materials have been proposed and debated in the past decades *inter alia* [113,114,115,116]. Many researchers have devised their own procedures for evaluating treatments, using a range of tests and techniques that are available to them and tailored to specific case studies. Although national assessment protocols exists (e.g., Normal UNI 11551-1:2014, Methodology for the Evaluation of a Cleaning Method; Part 1: Analytical protocol aimed examination of the potential harmfulness), unified international standard methods are still not available. European standards for treatment assessments are currently under consideration by the European Technical Committee 346. Confirmation of the safety of these control approaches will further favor their diffusion in a wide range of applications, leading to the development of standardized protocols and cost-to-benefit evaluations. Another issue related to research concerning the control of cultural heritage microbial deterioration relates to the fact that many of the methods for assessing treatment success are often arbitrary, qualitative, and non-replicable. There are no standardized methods to measure the efficacy of a control strategy, and, more importantly, it is not possible to compare the results obtained by different authors. Definitely, a unified and globally accepted approach by the scientific community would result in best practice guidelines for conservators.

Microorganisms do not always cause chemical or physical modification to intrinsic heritage property, which would lead to a loss of value or to impairment of use. This phenomenon can be the case for microbial discoloration, which implies only a change to the heritage surface color in one to three of the color parameters: hue, value, and chroma [117]. Finally, in the case of lithic materials, the growth of biofilms can lead to the formation of a stable layer protecting the surface from further weathering. Such bioprotection would have the additional advantage of being more compatible with the preservation of heritage surfaces in comparison to traditional protective coatings. More and more studies in recent conservation literature have investigated the role of biofilms on heritage surfaces, taking into account both biodeterioration and bioprotection processes [118,119]. Thus, in the near future, it will be vital to have techniques and protocols to clearly understand when we are dealing with biodeterioration, a mere aesthetic alteration, or bioprotection. However, we also want to highlight here that control strategies are not always the solution, even if biodeterioration is in place. This is a very important concept in line with the guidelines by ICOMOS (International Council on Monuments and Sites) [120] that “*No actions should be undertaken without demonstrating that they are indispensable*”.

## Figures and Tables

**Table 1:** Advantages and drawbacks of the principal control methods.

	Control Strategy	Advantages	Drawbacks
**Chemical Methods**	Traditional chemical biocides	Wide variety of compounds available on the market. Cheap and generally easy to apply. Effective against a broad range of microorganisms. Application in remote areas.	Toxic for the operators and the environment. The long-term effectiveness is very low. Often not selective against specific biodeteriogens. Promotion of biocide-resistant communities. Possible modification of biofilm structures favoring the growth of more harmful biodeteriogens. Repeated use may damage the heritage material.
	Nanoparticles	Wide variety of compounds available on the market. Easy to apply. Effective at very low concentrations. Application in remote areas.	Possible toxic effects on the operators and the environment. Not selective against specific biodeteriogens. Promote biocide-resistant communities. Lack of experiments discussing the interference with the heritage materials. Costly.
**Physical Methods**	Mechanical removal	Efficient method on surfaces with good state of conservation. Instant results. Do not require the use of toxic compounds. Do not generate toxic products.	The long-term effectiveness is very low. Repeated use may damage the heritage material. Biological contaminants can be pushed deep into the heritage material and spread in the environment.
	UV-C irradiation	Do not introduce any harmful chemicals to humans, to environment, or to the heritage material. Do not generate any toxic residual element in the environment. Simple application.	Repeated use may damage organic heritage material such as wood, leather, parchment, and textiles. Low penetration in substrates and in very thick biofilms. Not selective against specific biodeteriogens. Limited application in remote areas.
	Gamma radiation	Do not introduce any harmful chemicals to humans, to environment, or to the heritage material. High penetration in substrates and in very thick biofilms.	Repeated use may damage organic heritage material such as wood, leather, parchment, and textiles. Require specialized staff. Applications are limited to artworks of limited size. No longer possible to carry out radioluminescence dating after irradiation. Limited application in remote areas. Costly.
	Laser cleaning	Controllable, selective, contactless, and environmentally friendly. Do not introduce any harmful chemicals to humans, to environment, or to the heritage material. Instant results with highly localized effect. Do not generate any toxic residual elements in the environment.	Repeated use may damage the heritage material. Not selective against specific biodeteriogens. Limited application in remote areas. Required specialized staff. Costly.
	Heat shocking, microwaves, and dry ice treatment	Instant results with highly localized effect. Do not require the use of toxic compounds. Do not generate toxic products.	Microwaves and dry ice treatment equipment complicated to transport and apply, require continued access to energy supply, and are costly. Hazardous to handle. Not selective against specific biodeteriogens. Repeated use may damage some fragile surfaces. Limited application in remote areas. Costly.
**Biological Methods**	Biocidal treatments with compounds of natural origin	Generally safer for human beings and greener for the environment than traditional biocides. Generally easy to apply. Effective against a broad range of microorganisms. Application in remote areas.	The extract composition depends on the harvesting season, geographical location, and other agronomic factors. Only few products available on the market. Not selective against specific biodeteriogens. Lack of experiments discussing the interference of the natural compounds with the heritage materials.
	Other biological methods	Harmless for humans and the environmental health. Relatively easy to set up and improve. Effective against a broad range of microorganisms. Selective for the target microorganism. Application in remote areas.	Lack of experiments discussing the interference with the heritage materials. Lack of experiments assessing the persistence over time of the treatment. Costs evaluation needs to be done.
